# Assessment of domestic violence and its associated factors among ever-married reproductive-age women in Cameroon: a cross-sectional survey

**DOI:** 10.1186/s12905-022-01907-7

**Published:** 2022-10-01

**Authors:** Bezawit Mulat, Menen Tsegaw, Dagmawi Chilot, Kegnie Shitu

**Affiliations:** 1 Department of Human Physiology, School of Medicine, College of Medicine and Health Sciences, University of Gondar, Gondar, Ethiopia; 2 Department of Health, Education and Behavioral Sciences, Institute of Public Health, College of Medicine and Health Sciences, University of Gondar, Gondar, Ethiopia; 3 Department of Public Health, College of Medicine and Health Sciences, Ambo University, Ambo, Ethiopia

**Keywords:** Cameroon, Domestic violence, Married women

## Abstract

**Background:**

Domestic violence (DV) against women is a global issue that affects women in all countries and is a significant contributor to their poor health. Women who have experienced DV, in particular, suffer from the gynecological, nervous system, and stress-related issues. Despite such devastating implications, there is a paucity of data on the prevalence of DV and its associated factors among married reproductive-age women in Cameroon.

**Method:**

The data were analyzed by using STATA version 14 from a demographic and health survey conducted in Cameroon in 2018. Both bivariable and multivariable logistic regression analyses were done. Statistical significance was determined using a p-value of less than 0.05 and a 95% confidence range.

**Result:**

A total of 4,903 ever-married women were included in the study. The mean age ± standard deviation, of the participants was 31.3 (± 8.4) years with an age range of 15–49 years. The prevalence of DV among ever-married Cameroonian women was 30.5% with 95%CI (29.3%, 31.8%). Women’s educational attainment (higher educational level) (AOR = 0.58, 95% CI (0.37, 0.92), p value = 0.02), Husband's educational level (husbands who attended primary educational level (AOR = 1.30, 95% CI (1.01, 1.68), p value = 0.04), a woman who had media exposure regarding DV ( AOR = 0.83, 95% CI (0.0, 0.99), p value = 0.04), a woman whose husband drinks alcohol (AOR = 3.00, 95% CI (2.56, 3.53), p value < 0.001), and the region where the women reside (center without Yaoundé (AOR = 2.48, 95% CI (1.75, 3.52), p value < 0.001), west (AOR = 1.49, 95% CI (1.05, 2.11), p value = 0.02), South (AOR = 1.89, 95% CI (1.31, 2.72), p-value = 0.001), and Yaoundé (AOR = 1.65. 95% CI (1.14, 2.39), p value = 0.009) were factors that were significantly associated to DV committed by a husband in the previous 12 months against ever-married women.

**Conclusion:**

The present study revealed that the prevalence of DV among ever-married women in Cameroon is high. Women's educational level, husband's educational level, husband's alcohol drinking status, women’s exposure to mass-media about DV, and the region where the woman resides in the country were factors significantly associated with DV.

## Introduction

Domestic violence (DV) is defined as the intentional use of physical force or power, whether threatened or actual, against oneself, another person, or a group or community, which results in or has a high potential of resulting in injury or death [1]. DV is the intentional and frequently repeated physical, sexual, psychological, or financial abuse. The most common type of domestic violence is that which is perpetrated against women by their intimate partners [2]. DV against women is a worldwide problem that affects women in all countries and is a major contributor to their poorer health [3]. Unintended pregnancy, induced abortion, hemorrhage, HIV, and other sexually transmitted infections have all been linked to DV [4].

According to a WHO study on DV, intimate partner violence (IPV) is the most common form of violence in women’s lives, and women are more likely to be harmed at home than on the streets, with serious health effects [1]. Women who have been sexually and physically abused by intimate partners are more likely to suffer gynecological, nervous system, and stress-related problems [5].

DV occurs in all countries, but its prevalence varies greatly across the world and even within sub-Saharan Africa [5, 6]. For instance, it is 28.8% and 15% in Bangladesh and Thailand respectively [7, 8]. Moreover, in Africa 78.0% in somewhere in Ethiopia [9], 42.7% in Zimbabwe [10], 67.2% in north-central Nigeria [11], and 76.92% in Senegal [12]. Studies showed that sociodemographic factors like age, educational status of the women, educational status of the husband, husband drinking alcohol, and wealth index of the women are identified as factors that have a significant association with domestic violence [9, 10].

Domestic violence is not recognized as a specific crime in Cameroon and they don’t have a legal definition of domestic violence [13]. Moreover, there are no studies conducted on the assessment of DV in Cameroon by analyzing nationally representative data (DHS) data. Therefore, the present study is aimed to assess the prevalence and associated factors of domestic violence among ever-married reproductive-age Cameroonian women.

## Methods

### Study setting, data source, and study design

Cameroon is a country in western Africa that borders Central Africa. Cameroon is a triangular-shaped country bordered on the northwest by Nigeria, on the northeast by Chad, on the east by the Central African Republic, on the southeast by the Republic of Congo, on the south by Gabon and Equatorial Guinea, and on the southwest by the Atlantic Ocean [14]. Cameroon's population is currently projected to be 27,656,531 [15].

This cross-sectional study is employed based on the data from the 2018 Demographic and Health Survey (DHS) of Cameroon. The research participants are chosen using a two-stage stratified sampling technique. This data set (IR file) consists of information collected from all eligible women aged 15–49 years and the current study excludes unmarried women and employed with a total weighted sample of 4,903 ever-married reproductive-age women. An authorization letter for the use of this data was obtained from the DHS program and the dataset was downloaded from the DHS website www.measuredhs.com.

### Study variables

The outcome variable was the experience of DV among ever-married reproductive-age women in Cameroon. The variable was categorized into two categories: 1 = “experienced domestic violence” and 0 = “never experience domestic violence”. Physical violence plus emotional violence plus sexual violence constituted DV. In this study, the independent variables included were age religion, residence, educational status of women, educational status of the husband, current working (employment) status, wealth index, mass-media exposure, and behavioral factor( husbands’ alcohol drinking behavior).

### Operational definitions

*Domestic violence*: is defined as the presence of physical, emotional, or sexual violence, or a combination of all three [10].

*Physical violence* was defined as one or more intentional acts of physical aggression such as: pushing, slapping, throwing, hair pulling, punching, hitting, kicking, or burning, perpetrated with the potential to cause harm, injury, or death [16].

*Psychological/emotional violence* was defined as one or more acts, or threats of acts, including shouting, controlling, intimidating, humiliating, and threatening the victim [10, 16].

*Sexual violence* is defined as the use of force, coercion, or psychological intimidation to force a woman to engage in a sex act against her will, whether or not it is completed [16, 17].

### Data processing and analysis

Individual records (IR) files were used to extract data, which was then coded and transformed using STATA version 14 statistical software. To account for the differential chance of selection and non-response to the original survey, weighted samples were used for analysis. Bivariable binary logistic regression analysis was employed to identify factors that are eligible for multivariable binary logistic regression analysis at a p-value less than 0.2. Model fitness was checked with Hosmer and Lemeshow goodness fit test and it was fitted. The variance inflation factor (VIF) was also used to analyze multicollinearity across the explanatory components, and it was found to be within an acceptable range (1–4) [18]. The 95% confidence interval and a p value of 0.05 were applied to quantify statistical significance.

## Result

A total of 4,903 ever-married women were included in the study. The mean age of the participants with standard deviation (SD) was 31.3 (± 8.4) years with an age range of 15–49 years (Table 1).

**Table 1:** Sociodemographic characteristics of married women in Cameroon (n = 4903)

Variable	Category	Frequency	Percent
Age group (in years)	15–24	1126	23.0
	25–34	2152	44.0
	35–49	1625	33.0
Residence	Rural	2429	49.5
	Urban	2474	50.5
Religion	Christian	3302	67.4
	Muslim	1406	28.6
	Other	195	4.0
Educational status of a woman	No formal education	1330	27.0
	Primary	1552	31.7
	Secondary	1760	36.0
	Higher	262	5.3
Educational status of the husband	No formal education	905	18.4
	Primary	1327	27.0
	Secondary	1511	31.0
	Higher	394	8.0
	Don’t know	765	15.5
Wealth index	Poor	2037	41.5
	Middle	948	19.3
	Rich	1918	39.2
Current working status	Currently working	3478	29.1
	Currently not working	1426	70.9
Mass-media exposure	Yes	2804	57.8
	No	2099	42.8
Husband drinks alcohol	Yes	2253	46.0
	No	2650	54.0
A region where respondents reside	Adamawa	265	5.4
	Centre without Yaoundé	402	8.2
	Douala	553	11.3
	East	318	6.5
	Far north	929	19
	Littoral (without Yaoundé)	171	3.5
	North	699	14.3
	Northwest	335	6.9
	West	468	9.6
	South	179	3.7
	Southwest	92	1.9
	Yaoundé	492	10.0

Other (Animist, no religion, and other)

### Prevalence of domestic violence

In the current study, the prevalence of DV among ever-married Cameroonian women was 30.5% with 95%CI (29.3%, 31.8%). The proportion of DV was higher among age groups of women from 25 to 34 (32.7%) and 35–49 (30.3%) as compared to women found in the age groups of 18–24. Furthermore, domestic violence was higher among women whose husband drinks alcohol (43.2%).

### Prevalence of different forms of violence

Out of 4903 women involved in the study, 21.87%, 18.7%, and 6.5% of the participants experienced emotional, physical, and sexual violence by their husbands in the past 12 months respectively.

### Factors associated with domestic violence

In the bivariable logistic regression analysis, independent variables with a p value of less than 0.2 were passed to be included in the multivariable logistic regression analysis. Those variables were residence, religion, women’s educational level, current working status of a woman, husband’s educational level, women’s mass-media exposure to DV, husband’s alcohol drinking status, and the region where a woman resides. Based on multivariable binary logistic regression analysis the following explanatory variables had a statistically significant association with DV among ever-married reproductive-age women: Women's educational attainment (women who had a higher level of education (AOR = 0.58, 95% CI (0.37, 0.92), p value = 0.02), Husband’s educational level (husbands who attended primary educational level (AOR = 1.30, 95% CI (1.01, 1.68), p value = 0.04), women who had an exposure to mass-media about the DV (AOR = 0.83, 95% CI (0.70,0.99), p value = 0.04), women whose husband drinks alcohol (AOR = 3.00, 95% CI (2.56, 3.53), p value < 0.001), and the region where the women resides (center without Yaoundé (AOR = 2.48, 95% CI (1.75, 3.52), p value < 0.001), west (AOR = 1.49, 95% CI (1.05, 2.11), p value = 0.02, South (AOR = 1.89, 95% CI (1.31, 2.72), p value = 0.001), and Yaoundé (AOR = 1.65, 95% CI (1.14, 2.39), p value = 0.009). The odds of DV was decreased by 42% among married woman who had a higher level of educational attainment. The odds of DV increased by 30% among women whose husband’s education is at a primary level (Table 2).

**Table 2:** Factors associated with domestic violence among ever-married women in Cameroon, (n = 4903)

Variable	Domestic violence		Bivariable analysis		Multivariable analysis	
	Yes (n = 1496)	No (n = 3407)	p value	COR	p value	AOR
	Frequency (30.5%)	Frequency (69.5%)				
*Age*^c^	31.3 (± 8.1)^µ^	31.2 (± 8.1)^µ^	0.55	1.03 (0.94, 1.11)		
*Residence*						
Urban	724 (29.3%)	1750 (70.7%)		1		1
Rural	772 (31.8%)	1657 (68.2%)	< 0.001	1.26 (1.12, 1.43)	0.09	1.16 (0.98, 1.36)
*Religion*						
Catholic	1139 (34.5%)	2163 (65.5%)		1		1
Muslim	302 (21.5%)	1103 (78.5%)	< 0.001	0.47 (0.39, 0.54)	0.46	1.08 (0.88, 1.34)
Other	55 (28.1%)	140 (71.9%)	0.43	0.87 (0.63,1.22)	0.81	1.04 (0.73, 1.50)
*Women’s educational level*						
No formal education	301 (22.6%)	1029 (77.4%)		1		1
Primary	525 (33.9%)	1026 (66.1%)	< 0.001	1.65 (1.38, 1.96)	0.93	0.98 (0.79, 1.25)
Secondary	625 (35.5%)	1135 (64.5%)	< 0.001	1.78 (1.50, 2.11)	0.48	1.10 (0.85, 1.42)
Higher	45 (17.3%)	217 (82.7%)	0.063	0.71 (0.49, 1.01)	0.02	0.58 (0.37, 0.92)
*Current working status of a woman*						
Currently working	1125 (32.3%)	2353 (67.7%)	< 0.001	1.46 (1.27, 1.68)	0.13	1.13 (0.96, 1.32)
Currently not working	371 (26.0%)	1054 (74.0%)		1		1
*Husband's educational level*						
No formal education	177 (19.6%)	727 (80.4%)		1		1
Primary education	456 (34.4%)	871 (65.6%)	< 0.001	2.05 (1.66, 2.53)	0.04	1.31 (1.01, 1.67)
Secondary education	534 (35.3%)	979 (64.7%)	< 0.001	1.96 (1.59, 2.40)	0.17	1.21 (0.93, 1.58)
Higher	87 (22.2%)	307 (77.8%)	0.80	1.04 (0.7,1. 41)	0.16	0.76 (0.52, 1.11)
Don’t know	242 (31.6%)	523 (68.4%)	< 0.001	1.89 (1.50, 2.39)	0.36	1.14 (0.86, 1.50)
*Wealth index*						
Poor	615 (30.2%)	1422 (69.8%)		1		
Medium	290 (30.5%)	658 (69.5%)	0.84	1.02 (0.86, 1.20)		
Rich	592 (30.8%)	1327 (69.2%)	0.65	1.03 (0.90, 1.18)		
*Mass-media exposure*						
Exposed	911 (32.5%)	1893 (67.5%)	0.16	1.09 (0.96, 1.24)	0.04	0.83 (0.70, 0.99)
Non exposed	586 (27.9%)	1514 (72.1%)		1		1
*Husband's alcohol drinking status*						
yes	973 (43.2%)	1280 (56.8%)	< 0.001	3.12 (2.74, 3.55)	< 0.001	3.00 (2.56, 3.53)
No	524 (19.7%)	2127 (80.3)		1		1
*Region*						
Adamawa	48 (18.4%)	217 (81.6%)		1		1
Center without Yaoundé	197 (49.0%)	205 (51.0%)	< 0.001	4.13 (3.02, 5.64)	< 0.001	2.48 (1.75, 3.52)
Douala	164 (29.8%)	388 (70.2%)	0.003	1.66 (1.18, 2.33)	0.41	1.18 (0.79, 1.74)
East	112 (35.1%)	206 (64.9%)	< 0.001	2.43 (1.77, 3.34)	0.08	1.37 (0.97, 1.94)
Far-north	177 (19.0%)	752 (81.0%)	0.62	1.08 (0.79, 1.48)s	0.42	0.87 (0.62, 1.21)
Littoral (without Douala)	39 (23.3%)	131 (76.7%)	0.07	1.39 (0.97, 1.99)	0.25	0.779 (0.53, 1.18)
North	208 (29.7%)	491 (70.3%)	0.002	1.64 (1.20, 2.23)	0.07	1.34 (0.97, 1.86)
North -west	122 (36.6%)	213 (63.4%)	< 0.001	2.46 (1.73, 3.49)	0.06	1.43 (0.97, 2.10)
west	176 (37.7%)	292 (62.3%)	0.001	2.26 (1.65, 3.11)	0.02	1.49 (1.05, 2.11)
South	67 (37.4%)	112 (62.6%)	< 0.001	2.75 (1.98, 3.82)	0.001	1.89 (1.31, 2.72)
South west	25 (26.8%)	67 (73.2%)	0.017	1.77 (1.11, 2.83)	0.41	1.24 (0.74, 2.08)
Yaoundé	159 (32.4%)	333 (67.6%)	< 0.001	2.03 (1.47, 2.82)	0.009	1.65 (1.14, 2.39)

N.B: Hosmer and Lemeshow model fitness (> 0.05) and the variance inflation factor within the acceptable range (1–4)

## Discussion

By examining Cameroon's most recent DHS data, this study investigated the prevalence and associated factors of DV among ever-married women. In the last 12 months. The prevalence of domestic violence among ever-married Cameroonian women was 30.5% with 95%CI (29.3%, 31.8%). And Women’s educational level, husband's educational level, women’s mass-media exposure to DV, husband’s alcohol drinking status, and the region where the women reside were factors that had a statistically significant association with DV among ever-married women in Cameroon.

The prevalence of DV in this study was significantly lower than those of previous studies done in Fagitalekoma, Woreda, Awi zone, Ethiopia, which found 78.0% [10], Zimbabwe (42.7%) [11], Nigeria (67.2%) [12], Senegal (56.92%) [13], and Egypt (40.8%) [19]. This disparity could be attributed to socio-demographic characteristics in some of the countries that encourage wife-beating behavior, and some studies assess the lifetime prevalence of DV. As a result, when compared to our study, those factors may increase the prevalence of DV in those countries. However, the results of the present study are higher than the study conducted in rural Nepal which is 23.1% [16]. This difference is possibly due to differences in sociodemographic characteristics of the population.

DV among ever-married women was affected by different socio-demographic characteristics of both the women and their husbands. DV was found to be strongly connected with women's educational status in the current study, with women with a higher level of education, having a lower risk of experiencing domestic violence by their husbands. This result is supported by studies conducted in Saudi Arabia [20] and Kenya [21]. According to Chenna Kal's study, education may help people overcome ignorance, develop moral ideas, and improve their character. According to the study, education is also a tool that improves people's thinking and judgment of what is right and wrong, which encourages women to fight violence [22]. Moreover, the results of the present study stated that women who had husbands with a primary level of education had a higher risk for domestic violence than husbands with a higher level of education. This outcome is in harmony with studies done in Nigeria, Nepal, and Turkey respectively [23–25]. This could be explained by husbands with lower educational status who may have a lower level of awareness about women's rights and legislation that states domestic violence. DV among married women has also had a significant association with their husband's alcohol drinking behavior. This result is supported by a study done in Ethiopia, Zimbabwe, and Nepal [9, 10, 24]. This is because, alcohol consumption has a direct impact on human physico-cognitive function, reducing self-control and making people less capable of negotiating a nonviolent resolution to conflict within relationships. This may lead to domestic violence [26]. Furthermore, there is a statistically significant association between DV and women’s exposure to mass-media about DV. That is, compared to their counterparts, women who had access to mass-media had a decreased probability of experiencing DV. This result is supported by a study done in India [27]. Finally, the present study also revealed that the region where the women reside in the country is also another factor that was significantly associated with domestic violence. This disparity in the prevalence of domestic violence among married women across different regions of Cameroon may be due to differences in the socioeconomic status of the people living in such divergent regions of the Country**.**

## Conclusion and recommendations

The present study revealed that the prevalence of domestic violence among ever-married women in Cameroon is high. Women’s educational level, husbands’ educational level, husband’s alcohol drinking behavior, women’s media exposure to DV, and the region where the woman resides in the country were factors significantly associated with DV. As a result, the government should devise a comprehensive program to minimize domestic violence, taking into consideration the study's findings and enabling women to protect their legal rights.

## Data Availability

All results-based data are available within the manuscript and anyone can access the data set online from www.measuredhs.com

## Abbreviations

AOR: Adjusted odds ratio
CI: Confidence interval
DV: Domestic violence
DHS: Demographic Health Survey
